# Supplementary material for: The Small RNA Universe of Capitella teleta
Source: Front Mol Biosci. 2022 Feb 25;9:802814. doi: 10.3389/fmolb.2022.802814 (PMC8915122; doi:10.3389/fmolb.2022.802814)
Supplement: Supplementary file 1 [file DataSheet1.ZIP › Supplement/known/CAPTEscaffold_20_2495.pdf]

Provisional ID	:	CAPTEscaffold_20_2495
Score total	:	24091.8
Score for star read(s)	:	3.9
Score for read counts	:	24081.2
Score for mfe	:	2.1
Score for randfold	:	1.6
Score for cons. seed	:	3
Total read count	:	47246
Mature read count	:	47121
Loop read count	:	0
Star read count	:	125

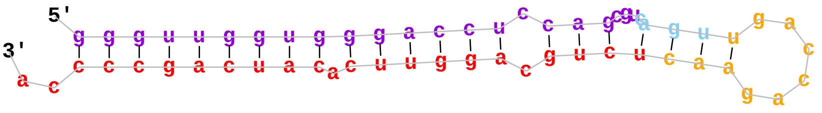

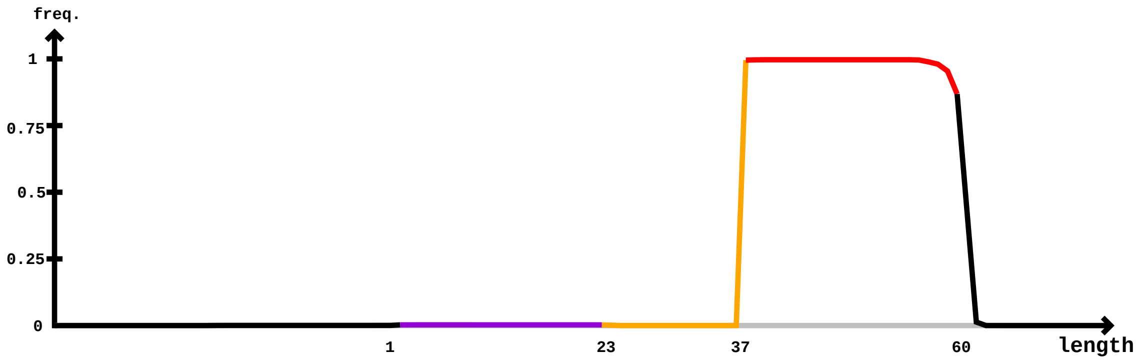

Star **Mature**

```
5'- cauugcugcccuugucgucaugcgcggcuccguugggguuggugggaccuccagcgucaguugaccagaacucugcagguucacaucagccccauggggucgcugccuu   -3'    obs
    cauugcugcccuugucgucaugcgcggcuccguugggguuggugggaccuccagcgucaguugaccagaacucugcagguucacaucagccccauggggucgcugccuu          exp
    cauugcugcccuugucgucaugcgcggcuccguugggguuggugggaccuccagcgucaguugaccagaacucugcagguucacaucagccccauggggucgcugccuu          known
    .....................((((((((((((.(((((((((((((((.(((....((((......))))))).)))))).)))))))))).)))))))))).))...   reads   mm   sample
    ..............ucgucaugcgcggcuccguu..........................................................................   3       0    seq
    ...............cgucaugcgcggcuccguu..........................................................................   18      0    seq
    ................gucaugcgcggcuccguu..........................................................................   1       0    seq
    ....................ugcgcggcuccguuggguuggu..................................................................   3       0    seq
    ................................uuggguuggugggaccucca........................................................   1       0    seq
    ..................................ggguuggugggaccuccagcg.....................................................   2       0    seq
    ..................................ggguuggugggaccuccagcgu....................................................   114     0    seq
    ..................................ggguuggugggaccuccagcgA....................................................   1       1    seq
    ..................................gggGuggugggaccuccagcgu....................................................   1       1    seq
    ..................................ggguuggugggaccuccagAgu....................................................   1       1    seq
    ..................................ggguuggugggaccuccagcgG....................................................   1       1    seq
    ..................................ggguuggugggaccucUagcgu....................................................   1       1    seq
    ....................................guuggugggaccuccagc......................................................   2       0    seq
    ......................................uggugggaccuccagcgu....................................................   1       0    seq
    ....................................................................Ucucugcagguucacaucagcccc................   2       1    seq
    .....................................................................cucugcagguucacaucagcccca...............   6       0    seq
    .....................................................................cucugcagguucacaucagccccau..............   2       0    seq
    ......................................................................ucugcagguucacaucag....................   41      0    seq
    ......................................................................Acugcagguucacaucag....................   1       1    seq
    ......................................................................ucAgcagguucacaucagc...................   1       1    seq
    ......................................................................Acugcagguucacaucagc...................   1       1    seq
    ......................................................................ucugcagguuAacaucagc...................   1       1    seq
    ......................................................................ucugcagguucacaucagc...................   293     0    seq
    ......................................................................ucugcagUuucacaucagc...................   1       1    seq
    ......................................................................ucugcagguucacaucagA...................   37      1    seq
    ......................................................................ucugcagguAcacaucagcc..................   1       1    seq
    ......................................................................ucugcagguucGcaucagcc..................   1       1    seq
    ......................................................................ucugcGgguucacaucagcc..................   1       1    seq
    ......................................................................ucugcaggCucacaucagcc..................   1       1    seq
    ......................................................................ucugcagguucacauUagcc..................   1       1    seq
    ......................................................................ucugcagguucacauAagcc..................   1       1    seq
    ......................................................................Gcugcagguucacaucagcc..................   1       1    seq
```

Star Mature

```
cauugcugcccuugucgucaugcgcggcuccguuggguugguggggaccuccagcgucaguugaccagaacucugcagguucacaucagccccauggggucgcugccuu
......................................................................ucugcagguucacaucagcc..................  404     0     seq
......................................................................ucugcagguucacaucagccU.................  54      1     seq
......................................................................ucugcagguucacaucagccc.................  1128    0     seq
......................................................................Ncugcagguucacaucagccc.................  1       1     seq
......................................................................ucugcagguucaUaucagccc.................  1       1     seq
......................................................................ucugcagCuucacaucagccc.................  2       1     seq
......................................................................ucugcagguucacaucagcUc.................  1       1     seq
......................................................................ucugcagguucacaucagcAc.................  1       1     seq
......................................................................Acugcagguucacaucagccc.................  5       1     seq
......................................................................ucAgcagguucacaucagccc.................  5       1     seq
......................................................................ucugcagguucGcaucagccc.................  1       1     seq
......................................................................ucugcagguucacaucagccA.................  11      1     seq
......................................................................ucugcagguuUacaucagccc.................  1       1     seq
......................................................................ucugcagguucacaCcagccc.................  1       1     seq
......................................................................ucuAcagguucacaucagccc.................  1       1     seq
......................................................................ucugcagguAcacaucagccc.................  1       1     seq
......................................................................Gcugcagguucacaucagccc.................  2       1     seq
......................................................................ucuAcagguucacaucagcccc................  1       1     seq
......................................................................ucugcaggAucacaucagcccc................  1       1     seq
......................................................................ucugUagguucacaucagcccc................  1       1     seq
......................................................................ucugcagguucacaAcagcccc................  3       1     seq
......................................................................ucugcagguucacaucagccUc................  1       1     seq
......................................................................ucugcagguAcacaucagcccc................  1       1     seq
......................................................................ucAgcagguucacaucagcccc................  7       1     seq
......................................................................ucugcagguucacaucagcccA................  14      1     seq
......................................................................ucugcagguucGcaucagcccc................  2       1     seq
......................................................................Ccugcagguucacaucagcccc................  1       1     seq
......................................................................ucugcagguucacaGcagcccc................  1       1     seq
......................................................................ucugcagguucacaucagcccc................  3900    0     seq
......................................................................ucugcagguuUacaucagcccc................  5       1     seq
......................................................................ucugcagguuAacaucagcccc................  4       1     seq
......................................................................ucCgcagguucacaucagcccc................  1       1     seq
......................................................................ucugAagguucacaucagcccc................  1       1     seq
......................................................................Gcugcagguucacaucagcccc................  3       1     seq
......................................................................ucugcagguucacauUagcccc................  4       1     seq
......................................................................ucugcagguucacaucGgcccc................  2       1     seq
......................................................................ucugcagguucacaCcagcccc................  1       1     seq
......................................................................ucugcagguucacaucagcccG................  1       1     seq
......................................................................ucugcaUguucacaucagcccc................  1       1     seq
......................................................................Acugcagguucacaucagcccc................  12      1     seq
......................................................................ucugcaggCucacaucagcccc................  1       1     seq
......................................................................ucugcagguucaUaucagcccc................  1       1     seq
......................................................................Ncugcagguucacaucagcccc................  2       1     seq
......................................................................ucugcagguucacUucagcccc................  1       1     seq
......................................................................ucugcagguucacaucaUcccc................  1       1     seq
......................................................................ucugcagguucacaucagccAc................  5       1     seq
......................................................................ucugcaAguucacaucagcccc................  3       1     seq
......................................................................ucugcagguucacaucagcccU................  72      1     seq
......................................................................ucugcagguucacaucagcAcca...............  24      1     seq
......................................................................ucugcaAguucacaucagcccca...............  22      1     seq
......................................................................ucuCcagguucacaucagcccca...............  1       1     seq
......................................................................ucugcagguucaGaucagcccca...............  3       1     seq
......................................................................Ccugcagguucacaucagcccca...............  6       1     seq
......................................................................ucugcaggAucacaucagcccca...............  8       1     seq
......................................................................ucugcagguucacaucagcccUa...............  37      1     seq
......................................................................ucugcagAuucacaucagcccca...............  6       1     seq
......................................................................ucugcagguucacaCcagcccca...............  1       1     seq
......................................................................ucugcaUguucacaucagcccca...............  6       1     seq
......................................................................ucugcagguucacaucagccccG...............  68      1     seq
......................................................................ucugUagguucacaucagcccca...............  3       1     seq
......................................................................uUugcagguucacaucagcccca...............  16      1     seq
......................................................................ucAgcagguucacaucagcccca...............  38      1     seq
......................................................................ucugcagguucacaucagcccca...............  33964   0     seq
......................................................................ucugcaggCucacaucagcccca...............  7       1     seq
......................................................................ucugcagguucacauAagcccca...............  7       1     seq
......................................................................ucugcagguucaAaucagcccca...............  7       1     seq
......................................................................ucugcagguAcacaucagcccca...............  38      1     seq
......................................................................ucugcagguucacaucagGccca...............  1       1     seq
......................................................................ucugcagguCcacaucagcccca...............  6       1     seq
......................................................................ucugcagguucacaucagccAca...............  19      1     seq
```

```
                                  Star                                 Mature
cauugcugcccuugucgucaugcgcggcuccguuggguuggugggaccuccagcgucaguugaccagaacucugcagguucacaucagccccauggggucgcugccuu
......................................................................uNugcagguucacaucagcccca...............   2      1      seq
......................................................................ucugcagCuucacaucagcccca...............   3      1      seq
......................................................................ucugcaCguucacaucagcccca...............   1      1      seq
......................................................................ucugcagguucacaucagcGcca...............   3      1      seq
......................................................................ucugcagguucaUaucagcccca...............   4      1      seq
......................................................................ucugcagguucacGucagcccca...............   3      1      seq
......................................................................Acugcagguucacaucagcccca...............   93     1      seq
......................................................................ucugcagguucacaucCgcccca...............   3      1      seq
......................................................................ucuAcagguucacaucagcccca...............   4      1      seq
......................................................................ucCgcagguucacaucagcccca...............   14     1      seq
......................................................................ucugcagguuGacaucagcccca...............   2      1      seq
......................................................................ucugcagguucGcaucagcccca...............   9      1      seq
......................................................................ucugcagguucacCucagcccca...............   1      1      seq
......................................................................ucGgcagguucacaucagcccca...............   2      1      seq
......................................................................ucugcagguucacaucagccccU...............   1100   1      seq
......................................................................ucugcagguucacaAcagcccca...............   18     1      seq
......................................................................ucugcagUuucacaucagcccca...............   4      1      seq
......................................................................ucugcagguucacaucagcccGa...............   5      1      seq
......................................................................ucugcagguucacaucagAccca...............   5      1      seq
......................................................................ucugAagguucacaucagcccca...............   7      1      seq
......................................................................ucugcGgguucacaucagcccca...............   6      1      seq
......................................................................ucugcagguucacaucagccUca...............   27     1      seq
......................................................................ucugcagguucUcaucagcccca...............   3      1      seq
......................................................................ucugcaggGucacaucagcccca...............   4      1      seq
......................................................................ucugcagguuAacaucagcccca...............   15     1      seq
......................................................................uAugcagguucacaucagcccca...............   4      1      seq
......................................................................Gcugcagguucacaucagcccca...............   42     1      seq
......................................................................ucugGagguucacaucagcccca...............   3      1      seq
......................................................................ucugcagguucacaucagcUcca...............   11     1      seq
......................................................................Ncugcagguucacaucagcccca...............   6      1      seq
......................................................................ucugcagguucacaucUgcccca...............   1      1      seq
......................................................................ucugcagguucacaucaCcccca...............   1      1      seq
......................................................................ucugcagguucCcaucagcccca...............   1      1      seq
......................................................................ucugcagguucacaucagUccca...............   6      1      seq
......................................................................ucugcagguucacauUagcccca...............   18     1      seq
......................................................................ucugcagguuUacaucagcccca...............   33     1      seq
......................................................................ucugcagguucacaucagccccC...............   91     1      seq
......................................................................ucugcagguucacaucagcccAa...............   14     1      seq
......................................................................ucugcagguucacaucGgcccca...............   8      1      seq
......................................................................ucugcagguucacauGagcccca...............   2      1      seq
......................................................................ucugcagguucacaucagccGca...............   3      1      seq
......................................................................ucugcagguucacUucagcccca...............   4      1      seq
......................................................................ucugcagCuucacaucagccccau..............   1      1      seq
......................................................................ucugcagguGcacaucagccccau..............   1      1      seq
......................................................................ucugcagguucGcaucagccccau..............   2      1      seq
......................................................................uAugcagguucacaucagccccau..............   1      1      seq
......................................................................ucugcagguucacaucagccUcau..............   2      1      seq
......................................................................ucugcagguuUacaucagccccau..............   5      1      seq
......................................................................ucugcagguucacaucagcUccau..............   1      1      seq
......................................................................ucugcagguucacGucagccccau..............   1      1      seq
......................................................................ucugcagguucacUucagccccau..............   1      1      seq
......................................................................Gcugcagguucacaucagccccau..............   4      1      seq
......................................................................ucugcagguucacaucagccccaA..............   465    1      seq
......................................................................ucugcagguucacaucagcccUau..............   7      1      seq
......................................................................ucugcagguuAacaucagccccau..............   2      1      seq
......................................................................ucugcagguucacCucagccccau..............   1      1      seq
......................................................................ucugcagguucaUaucagccccau..............   1      1      seq
......................................................................Acugcagguucacaucagccccau..............   8      1      seq
......................................................................ucugcagguucacaucagccccCu..............   7      1      seq
......................................................................ucugcagguucacaucagccGcau..............   1      1      seq
......................................................................ucugcagguucacauUagccccau..............   3      1      seq
......................................................................ucugcagguucacaucagGcccau..............   1      1      seq
......................................................................ucugcaAguucacaucagccccau..............   1      1      seq
......................................................................ucugcaggAucacaucagccccau..............   3      1      seq
......................................................................ucugcagguucacaucagccAcau..............   2      1      seq
......................................................................ucugcagguucacaucagccccau..............   3826   0      seq
......................................................................ucugcagguAcacaucagccccau..............   2      1      seq
......................................................................ucugcagguucacaucaAccccau..............   1      1      seq
......................................................................uUugcagguucacaucagccccau..............   4      1      seq
......................................................................ucugcagguucacaucagcAccau..............   1      1      seq
```

Star **Mature**

cauugcugcccuugucgucaugcgcggcuccguuggguuggugggaccuccagcgucaguugaccagaacucugcagguucacaucagccccauggggucgcugccuu			
....ucugcagguucacaucagAcccau..............	1	1	seq
....ucugcaCguucacaucagccccau..............	1	1	seq
....Ncugcagguucacaucagccccau..............	2	1	seq
....ucugcagguCcacaucagccccau..............	1	1	seq
....ucugcagguucacaucagcccAau..............	2	1	seq
....ucugcagguucacaucagccccaG..............	27	1	seq
....ucugcagguucacaAcagccccau..............	1	1	seq
....ucugcGgguucacaucagccccau..............	4	1	seq
....ucugcagguucacaucagccccUu..............	15	1	seq
....ucugcagguucacaucagccccaC..............	80	1	seq
....ucAgcagguucacaucagccccau..............	2	1	seq
....ucuAcagguucacaucagccccau..............	2	1	seq
....ucugcagguucacauAagccccau..............	1	1	seq
....ucugcagguucacaucagccccauC.............	15	1	seq
....ucugcagguucacaucagccccauA.............	34	1	seq
....ucugcagguucacaucagccccaAg.............	17	1	seq
....ucugcagguucacaucagccccauU.............	548	1	seq
....ucugcagguucacaucagccccauCg............	1	1	seq
....ucugcagguucacaucagccccaGggg...........	3	1	seq
....cugcagguucacaucagcccc................	3	0	seq
....cugcagguucacaucagcccca...............	29	0	seq
....cugcagguucacaucagccccC...............	1	1	seq
....cugcagguucacaucagccccCu..............	1	1	seq
....cugcagguucacaucagccccaA..............	1	1	seq
....cugcagguucacaucagccccau..............	12	0	seq
....cugcagguucacaucagccccaC..............	1	1	seq
....cugcagguucacaucagccccauU.............	1	1	seq
....cugcagguucacaucagccccauA.............	1	1	seq
....ugcagguucacaucagcccc................	1	0	seq
....ugcagguucacaucagccccU...............	4	1	seq
....ugcagguucacaucagcccca...............	11	0	seq
....ugcagguucacaucagccccau..............	1	0	seq
....gcagguucacaucagccccauggggucgcug....	1	0	seq
....agguucacaucagcccca...............	1	0	seq
